# Nostocyclopeptides as New Inhibitors of 20S Proteasome

**DOI:** 10.3390/biom11101483

**Published:** 2021-10-08

**Authors:** Anna Fidor, Katarzyna Cekała, Ewa Wieczerzak, Marta Cegłowska, Franciszek Kasprzykowski, Christine Edwards, Hanna Mazur-Marzec

**Affiliations:** 1 Division of Marine Biotechnology, Institute of Oceanography, University of Gdańsk, Marszałka Józefa Piłsudskiego 46, PL-81378 Gdynia, Poland; anna.fidor@phdstud.ug.edu.pl; 2 Department of Biomedical Chemistry, Faculty of Chemistry, University of Gdańsk, Wita Stwosza 63, PL-80308 Gdańsk, Poland; jedrzejewskakatarzyna.92@gmail.com (K.C.); ewa.wieczerzak@ug.edu.pl (E.W.); franciszek.kasprzykowski@ug.edu.pl (F.K.); 3 Institute of Oceanology, Polish Academy of Sciences, Powstańców Warszawy 55, PL-81712 Sopot, Poland; mceglowska@iopan.pl; 4 School of Pharmacy and Life Sciences, Robert Gordon University, Aberdeen AB10 7GJ, UK; c.edwards@rgu.ac.uk

**Keywords:** 20S proteasome inhibitors, cyanobacteria, *Nostoc*, nostocyclopeptides

## Abstract

Nostocyclopeptides (Ncps) are a small class of bioactive nonribosomal peptides produced solely by cyanobacteria of the genus *Nostoc*. In the current work, six Ncps were isolated from *Nostoc edaphicum* strain CCNP1411. The bioactivity of these compounds was tested in vitro against 20S proteasome, a proteolytic complex that plays an important role in maintaining cellular proteostasis. Dysfunction of the complex leads to many pathological disorders. The assays indicated selective activity of specific Ncp variants. For two linear peptide aldehydes, Ncp-A2-L and Ncp-E2-L, the inhibitory effects on chymotrypsin-like activity were revealed, while the cyclic variant, Ncp-A2, inactivated the trypsin-like site of this enzymatic complex. The aldehyde group was confirmed to be an important element of the chymotrypsin-like activity inhibitors. The nostocyclopeptides, as novel inhibitors of 20S proteasome, increased the number of natural products that can be considered potential regulators of cellular processes.

## 1. Introduction

The 26S proteasome is a large (2.4 MDa), multifunctional and ATP-dependent enzymatic complex with chymotrypsin-like (CT-L), trypsin-like (T-L), and caspase-like (C-L) activities [1,2,3,4]. In eukaryotic organisms, it recognizes and degrades proteins with covalently attached ubiquitin (8.5 kDa protein) [5,6]. The 26S proteasome is composed of a 20S barrel-shaped core particle (700 kDa) responsible for proteolytic activity and one or two 19S (890 kDa) regulatory subunits with ubiquitin-binding sites [3,4,7]. The 20S proteasome also occurs as a free complex that degrades proteins in the ubiquitin-independent pathway [8,9]. In humans, the dysfunction of this proteolytic machinery leads to changes in protein profile and, ultimately, to serious health problems. Therefore, proteasome regulators are explored as promising therapeutic agents for a range of diseases (e.g., cancer, autoimmune disorders, inflammation, malaria) [10,11,12]. The majority of the known 20S proteasome inhibitors belongs to peptide-based structures such as peptide aldehydes, boronates, epoxyketones, or peptide vinyl sulfones [13,14,15]. Some of the active compounds are of natural origin. Leupeptin, isolated from several strains of Gram-positive bacteria of the order *Actinomycetales*, inhibits T-L activity of the 20S proteasome [16]. Tyropeptin A, a peptide aldehyde produced by the soil *Streptomycetales* of the genus *Kitasatospora,* strain MK993-dF2, inhibits mainly CT-L activity [17,18]. Marine fungus *Peicillium fellutanum* is a producer of fellutamide B, a strong inhibitor of CT-L activity (IC_50_ 9.4 nM) with mild effects on T-L (IC_50_ 2.0 μM) and C-L (IC_50_ 1.2 μM) activities [19]. The proteasome inhibition within the nanomolar to the micromolar range of IC_50_ was also documented for metabolites isolated from cyanobacteria *Symploca* sp., *Scytonema hofmannii,* and *Nostoc*.

In our preliminary studies, fractions from *Nostoc edaphicum* CCNP1411 containing nostocyclopeptides (Ncps) inhibited the chymotrypsin-like activity of the 20S proteasome. Ncps constitute a small group of nonribosomal peptides solely produced by cyanobacteria of the genus *Nostoc*. The biological activity of the peptides was reported in several studies. According to Golakoti et al. [20], Ncp-A1 and Ncp-A2 have cytotoxic activity against human colorectal adenocarcinoma (LoVo) and human nasopharyngeal (KB) cell lines (IC_50_ ca. 1 μM). Another nostocyclopeptide variant, Ncp-M1, was shown to inhibit the transport of toxic microcystin-LR and nodularin into hepatocytes by blocking organic anion transporter polypeptides, OATP1B1, and OATP1B3. These polypeptides are also overexpressed in cancer cells [21,22]. The role of Ncp-M1 and its analogs as antitumor agents and as tools to study membrane transport was proposed [21,23,24].

Given the pharmaceutical potential of Ncps, the recognition of their action on different cellular targets is important. In the current work, the effects of Ncps on the 20S proteasome were explored. To determine structure-activity relationship, six different Ncps isolated from the Baltic cyanobacterium *N. edaphicum* CCNP1411 were tested, including linear and cyclic Ncp variants.

## 2. Materials and Methods

### 2.1. Organism, Extraction, and Isolation of Compounds

*Nostoc edaphicum* strain CCNP1411 (GenBank Accession Number: PRJNA638531) was isolated from the Gulf of Gdańsk, southern Baltic Sea. The cyanobacterium was grown in a Z8 medium enriched with NaCl [25]. The culture was kept in 2 L flasks at 22 ± 1 °C and light of 5–10 µmol photons m^−2^ s^−1^. After three weeks, the biomass was harvested using a nylon net (mesh size 25 µm).

The freeze-dried biomass of CCNP1411 (20 g) was homogenized and extracted four times with 75% methanol (MeOH) in MilliQ water (4 × 150 mL) by vortexing for 30 min. The extracts were centrifuged at 12,000× *g* for 15 min at 4 °C. Combined supernatants were diluted with MilliQ water to achieve the final concentration of MeOH < 10%. Isolation of Ncps was performed using the HPLC system (Shimadzu Corporation, Kyoto, Japan). During all chromatographic runs, the absorbance was monitored at 210 nm and 270 nm. The diluted sample was loaded onto a preconditioned 120 g SNAP KP-C18-HS column (100 Å, 30 µm) (Biotage, Uppsala, Sweden). The flash chromatography was performed with a mobile phase consisting of MilliQ water (phase A) and 100% MeOH (phase B) using a step gradient from 10 to 100% B over 180 min (flow rate 12 mL min^−1^). The volume of the fractions collected was 40 mL. Ncps-containing fractions were pooled, concentrated, and separated on Jupiter Proteo C12 column (250 × 21.2 mm; 90 Å; 4 µm) (Phenomenex, Torrance, CA, USA). The mobile phase was composed of 5% CH_3_CN in MilliQ water (phase A) and 100% CH_3_CN (phase B), both containing 0.1% of formic acid (flow rate 12 mL min^−1^). The chromatographic run (15–100% B) took 110 min, and 2 mL fractions were collected. All fractions containing Ncps mixture were combined, concentrated, and subjected to further separation under modified conditions. The gradient started at 15% B and for the first hour the content of phase B increased by 1% every 15 min up to 19% B. This concentration was maintained for the next 20 min, and then within 30 min, it increased linearly to 100% B. Pure Ncps (Ncp-A2 and Ncp-E4-L) were present in fractions eluted in the range 15–25% of phase B.

The remaining fractions containing Ncps were pooled, and the other four individual peptides were isolated using an analytical Agilent HPLC 1200 Series system (Agilent Technologies, Santa Clara, CA, USA) with a diode array detector (DAD) operating at 210 and 270 nm. Ncp-A1 was isolated using Jupiter Proteo C12 column (250 × 4.6 mm, 90 Å, 4 µm) (Phenomenex, Torrance, CA, USA), while Ncp-A2-L, Ncp-E2, and its linear analog Ncp-E2-L, were isolated on Zorbax Eclipse XDB-C18 column (4.6 × 150 mm; 80 Å; 5 µm) (Agilent Technologies, Santa Clara, CA, USA). During all analytical chromatographic runs, the same mobile phase (at flow rate 0.5 mL min^−1^) was used as for the preparative separations. Ncps were eluted when the mobile phase contained 16–43% B. Identification and purity of peptides in individual fractions and subfractions were achieved by LC-MS/MS analysis at each purification step. Analyses were carried out using Agilent 1200 HPLC (Agilent Technologies, Santa Clara, CA, USA) coupled to a QTRAP5500 triple-quadrupole/linear ion trap mass spectrometer (Applied Biosystems MDS Sciex, Concord, ON, Canada), as previously described [26]. Chromatographic separation was performed on a Zorbax Eclipse XDB-C18 column (4.6 × 150 mm; 80 Å; 5 µm) using gradient elution with the same mobile phase as for the HPLC-DAD analyses. The mass spectrometer operated under the positive Turbo Ion Spray ionization mode (5.5 kV, 550 °C). Tandem mass spectra were acquired at collision energy 60 V.

### 2.2. NMR Analysis

The 1D ^1^H NMR and 2D homo- and heteronuclear NMR (COSY, TOCSY, ROESY, HSQC, and HMBC) were acquired with the application of Bruker Avance III spectrometers, 500 MHz and 700 MHz (Bruker, Billerica, MA, USA). Spectra were recorded in H_2_O:D_2_O (9:1). NMR data were processed and analyzed by TopSpin (Bruker, Billerica, MA, USA) and SPARKY software (3.114, Goddard and Kneller, freeware https://www.cgl.ucsf.edu/home/sparky).

### 2.3. Human 20S Inhibition Assay

The 20S proteasome inhibition assay was performed following the procedure of Czerwonka et al. [27]. Human 20S proteasome (h20S) isolated from erythrocytes was used. Latent h20S was activated with 0.01% SDS (sodium dodecyl sulfate). The final concentration of the proteasome was 1 µg mL^−1^ (1.4 nM). The fluorogenic substrates, Suc-LLVY-AMC, Boc-LRR-AMC, and Z-LLE-AMC, were used as probes in the chymotrypsin-like, trypsin, and caspase-like activity assays, respectively, at a final concentration of 100 µM. Stock solutions of Ncps (10 mM) were prepared in dimethyl sulfoxide (DMSO) and were tested in the concentration range of 5 to 50 μM. The content of DMSO never exceeded 3% of the final reaction volume. The assays were performed in a 96-well plate in 50 mM TrisHCl, pH 8.0, at 37 °C. The percentage of the substrate hydrolysis was measured by the amount of the released AMC (aminomethyl coumarin) using Tecan Infinite M200 Pro (λ = 380–460 nm) spectrofluorimeter (Tecan Trading AG, Männedorf, Switzerland). The fluorescence measurements were performed at 2-min intervals for 60 min. The activity of h20S in the presence of isolated Ncps was calculated in relation to the control (DMSO). The known proteasome inhibitor PR11 [28] was used to ensure the correctness of the assay. The peptide at the final concentration of 0.2 µM decreases the relative CT-L activity of the h20S to 6% of the control.

## 3. Results and Discussion

**Figure 1:**
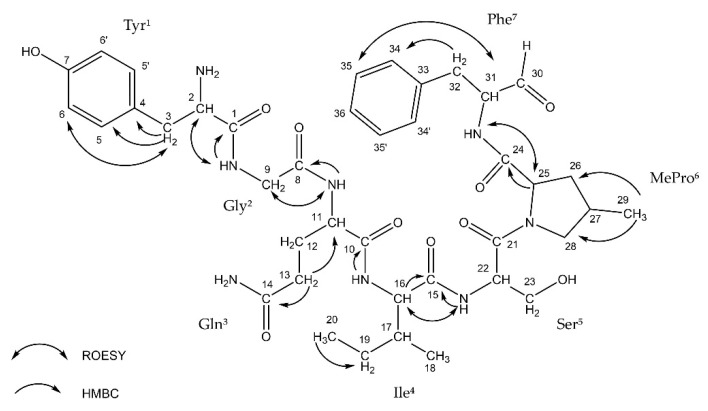
ROESY and HMBC correlations in nostocyclopeptide Ncp-A2-L.

Thus far, the presence of Ncps was reported in five strains of *Nostoc* isolated from different habitats [20,23,29,30,31]. This includes two Baltic strains: XSPORK 13A producing the cyclic Ncp-M1 [23] and CCNP1411 producing 10 other Ncps variants [31]. The putative structures of the five linear and five cyclic Ncps variants produced by CCNP1411 were elucidated based on mass spectra fragmentation patterns [31]. Two of the cyclic forms, Ncp-A1 and Ncp-A2, enclosed by imino linkage between the *N*-terminal amine group of conserved Tyr and *C*-terminal aldehyde group of Leu or Phe, were previously identified in *Nostoc* sp. ATCC53789 isolated from lichen [20]. In position 6 of the Ncps from CCNP1411, 4-methylproline (MePro) or Pro is present, while Ile or Val is in position 4 (Figure 1). In the current study, we were able to isolate 6 out of 10 Ncps produced by CCNP1411 (Table 1): three cyclic variants (Ncp-A1, Ncp-A2, and Ncp-E2), two linear aldehyde forms of the cyclic variants (Ncp-A2-L and Ncp-E2-L), and the six-amino acid peptide Ncp-E4-L lacking the aldehyde group in the *C-*terminus. In the case of four other Ncps produced by CCNP1411 (Ncp-A1-L, Ncp-E1, Ncp-E1-L, Ncp-E3), their purity and/or quantities were not sufficient for inclusion in the study. Ncp-A2-L (Figure 1) was the only variant obtained in sufficient amounts for NMR analyses. The ^1^H NMR spectrum of Ncp-A2-L displayed a typical pattern of a peptide. The COSY, TOCSY, and HMBC experiments allowed for the identification of the residues in Ncp-A2-L as Tyr, Gly, Gln, Ile, Ser, MePro, and phenylalaninal (Phe-H) (Table 2, Figure 1, Figures S1–S5). The amino acid sequence was confirmed by TOCSY data. The presence of two aromatic amino acid residues was recognized by the signals occurring in the aromatic region of the spectrum (δ_H_ 6.78–7.26 ppm). One of them was identified as tyrosine-based on the AA’BB’ spin system between the aromatic protons (Tyr-H5/5′ and Tyr-H6/6′, JH, H = 8.0 Hz). The second aromatic residue was identified as phenylalanine based on the TOCSY interaction between 34, 35, and 36 protons and the HMBC correlation from two diastereotopic methylene protons 32a (δ_H_ 2.57 ppm) and 32b (δ_H_ 2.98 ppm) to the aromatic 34/34′ carbons (Figures S3 and S5). The 4-methyl group of the proline residue was identified based on the ^1^H NMR doublet signal at δ 0.82 ppm (protons 29) and the HMBC correlation between the methyl protons with 26 (δ_C_ 37.3 ppm) and 28 (δ_C_ 55.0 ppm) carbons (Figures S1 and S5). The signal at δ_H_ 9.46 ppm was assigned to phenylalaninal aldehyde proton. The occurrence of the studied compound in the linear form was further confirmed by the lack of the ROESY correlation between tyrosine and phenylalanine residues.

**Figure 2:**
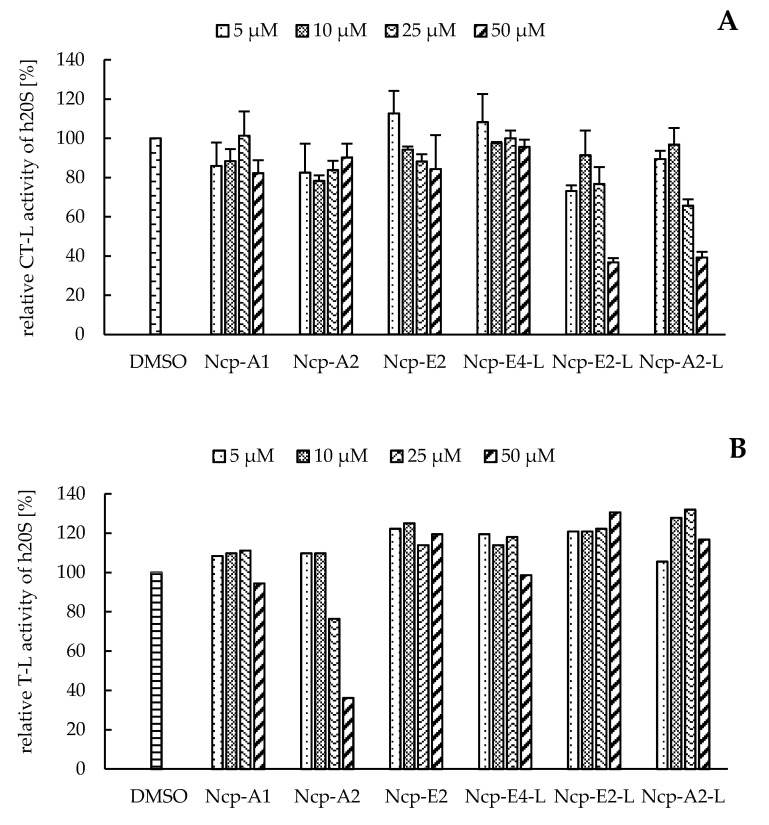

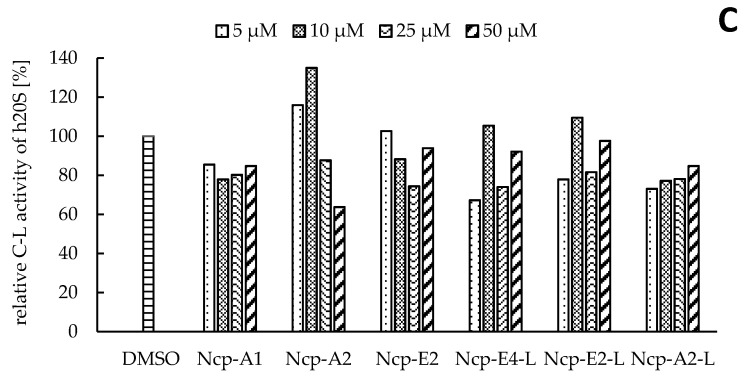
The effects of cyclic and linear (L) nostocyclopeptides (Ncps) on the CT-L (**A**), T-L (**B**), and C-L (**C**) activities of the human 20S proteasome. DMSO was used as a control, and PR11 was used to ensure the correctness of the assay. In the CT-L activity assay, all Ncp variants were tested in triplicate.

In our preliminary studies with the application of the human 20S proteasome, the Ncp-containing fractions of CCNP1411 inhibited CT-L activity at micromolar concentrations. In the current work, to unequivocally state which of the cyanobacterial metabolites are responsible for this activity, the six isolated Ncps were assayed. For three cyclic Ncps (Ncp-A1, Ncp-A2, Ncp-E2) and the six-amino acid linear variant without an aldehyde group (Ncp-E4-L), no effects on CT-L activity of the human 20S proteasome were observed (Figure 2A). This activity was inhibited only by two linear peptide aldehydes, Ncp-A2-L and Ncp-E2-L, applied at 50 µM (Figure 2A). As the two Ncps differ in position 6 (Pro/MePro) and the *C*-terminal amino acid (Leu/Phe), it can be concluded that these residues do not affect the CT-L activity. Nostocyclopeptide Ncp-E2-L, as well as the widely used synthetic proteasome inhibitor MG-132 [32,33], contain the aldehyde group on *C*-terminal Leu. The potent activity of MG-132 (IC_50_ 0.11 µM) [34] was attributed to the formation of the hemiacetal covalent bond between the aldehyde group of *C*-terminal Leu and the hydroxyl group of Thr1 present in the active site of the proteasome [35]. Another bioactive linear nostocyclopeptide from CCNP1411, Ncp-A2-L, also has the *C*-terminal amino acid aldehyde (Phe), which again confirms the importance of the aldehyde group for the CT-L inhibition [14,36]. Due to the limited amounts of the isolated Ncps, their effects on T-L and C-L activities were examined with no replications. In the assays, only the cyclic Ncp-A2 showed concentration-dependent inhibition of T-L activity (Figure 2B) and had weak effects on C-L activity (Figure 2C). The other Ncp variants had no clear effects on the two proteolytic sites.

The two linear Ncps, Ncp-A2-L and Ncp-E2-L, moderately decreased the CT-L activity (IC_50_ ca. 50 µM), compared with several known aldehyde-containing proteasome inhibitors [13]. However, this moderate potency of the Ncps is compensated by their high specificity. Unlike many other peptide aldehydes, which inhibit a wide range of proteases [13,36], the two Ncps interacted with the CT-L site but did not modify the T-L and C-L activities.

Among cyanobacteria metabolites, α,β-epoxyketones carmaphycin A and B, isolated from *Symploca* sp., were found to inhibit CT-L activity of the *Saccharomyces cerevisiae* 20S proteasome at low nanomolar concentrations [37]. The authors suggested that the sulfoxide/sulfone moieties in the methionine-derived residues of the inhibitor are crucial for the interaction with the enzyme complex. Nostodione A from *Scytonema hofmannii,* which inhibits CT-L activity (IC_50_ 50 μM), contains indole moiety fused with diketone system [38]. Other cyclic metabolites from cyanobacteria have demonstrated inhibitory effects against 20S complex. For example, scytonemide A from *S. hofmannii*, a cyclic peptide characterized by the presence of a unique imino linkage, inhibited catalytic activity of proteasome at IC_50_ 96 nM [39]. Krunic et al. [39] suggested that Gln residue contributed to structural conformation in this peptide, enabling optimal binding at the active site. On the other hand the presence of an imine enabled the formation of a covalent bond. *Nostoc*-derived (*Nostoc* sp. UIC 10022A) cylindrocyclophanes were active against the 20S proteasome in a wide range of activities (IC_50_ 2.2–100 μM). According to the authors, dichloromethyl moiety was crucial to achieving a higher level of inhibition [40].

The proteasome is an important drug target in a variety of diseases [10,11,12]. Currently, three proteasome inhibitors, approved by the American Food and Drug Administration (FDA), are clinically used for the treatment of multiple myeloma (MM) and mantle cell lymphoma (MVL) patients: bortezomib (Velcade) [41], carfilzomib (Kyprolis) [42] and ixazomib (Ninlaro) [43]. Unfortunately, despite the initial promising effects and high efficacy of the proteasome inhibitors in MM treatment, in many patients, resistance has developed. Moreover, some patients do not respond to this treatment, or the side effects of the drugs are too severe [44,45,46,47].

Currently, the application of proteasome inhibitors in the treatment of other diseases, e.g., in autoimmune disorders, inflammation, or malaria, is explored [4,48]. Further studies are also performed to better understand the effect of specific proteasome inhibitors on general protein homeostasis. In parallel, screening for novel agents with a potential therapeutic application as regulators of the 20S proteasome and other components of the ubiquitin-proteasome system is continued [49,50]. Regardless of the low potency, Ncps still can be considered as starting points for drug development. As in the case of many bioactive natural products, their activity and selectivity can be optimized by structural modifications so that the final compound can demonstrate a better therapeutic potential.

## 4. Conclusions

*Nostoc edaphicum strain* CCNP1411 produces 10 nostocyclopeptides, including cyclic and linear forms. In the current work, the effect of six isolated Ncps structural variants on the activity of the human 20S proteasome was examined. The results indicate the differences in the activity of the cyclic and linear Ncp variants and show their selectivity in interaction with the proteasome active sites. Two of the linear peptides, Ncp-A2-L and Ncp-E2-L, inhibited the CT-L activity of the enzymatic complex without any effect on T-L and C-L sites. On the other hand, the cyclic Ncp-A2 had an inhibitory effect on T-L activity. The study also confirmed the importance of an aldehyde group for the interaction with the active center responsible for the CT-L activity. This is the first report on the inhibitory effect of Ncps on the 20S proteasome, which is an important drug target in various diseases.

## Figures and Tables

**Table 1:** Structures of six nostocyclopeptide-variants isolated from *Nostoc edaphicum* CCNP1411 as pure compounds.

Peptide Name	Molecular Mass	Structure
Ncp-A1	756	[Tyr^1^+Gly^2^+Gln^3^+Ile^4^+Ser^5^+MePro^6^+Leu^7^]
Ncp-A2	790	[Tyr^1^+Gly^2^+Gln^3^+Ile^4^+Ser^5^+MePro^6^+Phe^7^]
Ncp-A2-L	808	Tyr^1^+Gly^2^+Gln^3^+Ile^4^+Ser^5^+MePro^6^+Phe-H^7^
Ncp-E2	742	[Tyr^1^+Gly^2^+Gln^3^+Ile^4^+Ser^5^+Pro^6^+Leu^7^]
Ncp-E2-L	760	Tyr^1^+Gly^2^+Gln^3^+Ile^4^+Ser^5^+Pro^6^+Leu-H^7^
Ncp-E4-L	676	Tyr^1^+Gly^2^+Gln^3^+Ile^4^+Ser^5^+MePro^6^

**Table 2:** Nuclear Magnetic Resonance (NMR) Spectroscopic Data for Ncp-A2-L (Tyr-Gly-Gln-Ile-Ser-MePro-Phe-H).

Residue	Position	δ_C_, type	δ_H_ (*J* in Hz)	ROESY	HMBC *^a^*
Tyr	1				
	2	169.9, C			
	3	54.6, CH	4.13, t (6.9, 6.9)	NH(1), 6	
	4	36.0, CH_2_	3.04, dd (7.3, 12.9)	6	
	5/5′	125.5, C			
	6/6′	130.9, CH	6.78, d (8.0)		2, 4, 5
	7	115.9, CH	7.04, d (8.0)	2, 3	
	NH_2_	155.3, C			
	OH				
Gly	8	170.7, C			
	9	42.4, CH_2_	3.84, m	NH(2)	
	NH(1)		8.46, t (5.6, 5.6)	2	1
Gln	10				
	11	173.1, C		NH(3)	
	12a	53.2, CH	4.30, m		10
	12b	27.2, CH_2_	1.88, m		
	13		1.99, m		
	14	31.1, CH_2_	2.26, t (7.3, 7.3)		11, 12, 14
	NH(2)	178.0, C		9	
	NH_2_		8.25, d (7.6)		8
Ile	15	173.4, C			
	16	58.2, CH	4.09, t (8.1, 8.1)	NH(4)	17
	17	36.0, CH	1.77, m		
	18	14.7, CH_3_	1.08, d (6.6)		
	19	24.6, CH_2_	1.32, m		
	20	10.0, CH_3_	0.79, t (7.3, 7.3)	22	17, 19
	NH(3)		8.21, d (6.8)	11	10
Ser	21				
	22	n.o.			
	23a	n.o.	4.59, m	20	
	23b	61.0, CH_2_	3.69, m		
	NH(4)		3.77, m		
	OH		8.31, d (5.1)	16	15
MePro	24	173.8, C			
	25	61.3, CH	4.15, dd (8.1, 9.3)		24
	26	37.3, CH_2_	2.13, m		
	27	33.1, CH	2.04, m		
	28a	55.0, CH_2_	2.84, t (10.5, 10.5)	NH(5)	
	28b		3.86, m		26
	29	15.2, CH_3_	0.82, d (6.6)		26, 28
Phe-H	30		9.46, s		
	31	n.o.	4.01, m	35	
	32a	55.5, CH	2.57, dd (10.6, 14.0)	34	34/34′
	32b	34.2, CH_2_	2.98, dd (4.0, 14.5)		
	33			32	
	34/34′	137.9, C	7.26, m		
	35/35′	129.5, CH	7.15, d (7.2)	31	
	36	128.7, CH	7.18, m		
	NH(5)	126.6, CH	7.46, d (9.3)	25	24

^a^ HMBC correlations are given from proton(s) stated to the indicated carbon atom.

## Data Availability

Not applicable.

## Supplementary Materials

The following are available online at www.mdpi.com/2218-273X/11/10/1483/s1. Figure S1: ^1^H NMR spectrum of Ncp-A2-L in H_2_O:D_2_O (9:1), Figure S2: COSY spectrum of Ncp-A2-L in H_2_O:D_2_O (9:1), Figure S3: TOCSY spectrum of Ncp-A2-L in H_2_O:D_2_O (9:1), Figure S4: HSQC spectrum of Ncp-A2-L in H_2_O:D_2_O (9:1), Figure S5: HMBC spectrum of Ncp-A2-L in H_2_O:D_2_O (9:1).
