# Supplementary material for: Nostocyclopeptides as New Inhibitors of 20S Proteasome
Source: Biomolecules. 2021 Oct 8;11(10):1483. doi: 10.3390/biom11101483 (PMC8533403; doi:10.3390/biom11101483)
Supplement: Supplementary file 1 [file biomolecules-11-01483-s001.zip › biomolecules-1385195-supplementary.pdf]

Supplementary Material: Nostocyclopeptides as new inhibitors of 20S proteasome

Anna Fidor [1], Katarzyna Cekała [2], Ewa Wieczerzak [2], Marta Cegłowska [3], Franciszek Kasprzykowski [2], Christine Edwards [4], Hanna Mazur-Marzec [1*]

[1] Division of Marine Biotechnology, Institute of Oceanography, University of Gdańsk, Marszałka Józefa Piłsudskiego 46, PL-81378 Gdynia, Poland; anna.fidor@phdstud.ug.edu.pl (A.F.); hanna.mazur-marzec@ug.edu.pl (H.M-M.)
[2] Department of Biomedical Chemistry, Faculty of Chemistry, University of Gdańsk, Wita Stwosza 63, PL-80308 Gdańsk, Poland; jedrzejewskakatarzyna92@gmail.com (K.C.); ewa.wieczerzak@ug.edu.pl (E.W.); franciszek.kasprzykowski@ug.edu.pl (F.K.)
[3] Institute of Oceanology, Polish Academy of Sciences, PowstańcówWarszawy 55, PL-81712 Sopot, Poland; mceglowska@iopan.pl
[4] School of Pharmacy and Life Sciences, Robert Gordon University, Aberdeen AB10 7GJ, UK; c.edwards@rgu.ac.uk (C.E.)
* Correspondence: hanna.mazur-marzec@ug.edu.pl (H.M-M.)

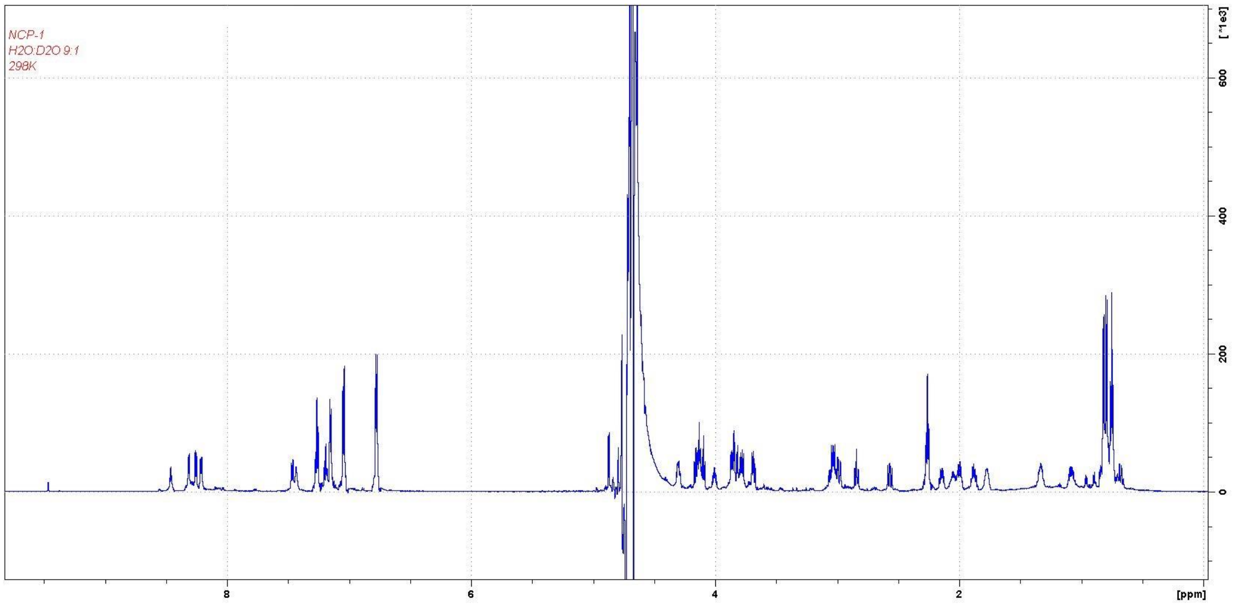

Figure S1: ^{1}H NMR spectrum of Ncp-A2-L in $H_2O:D_2O$ (9:1)

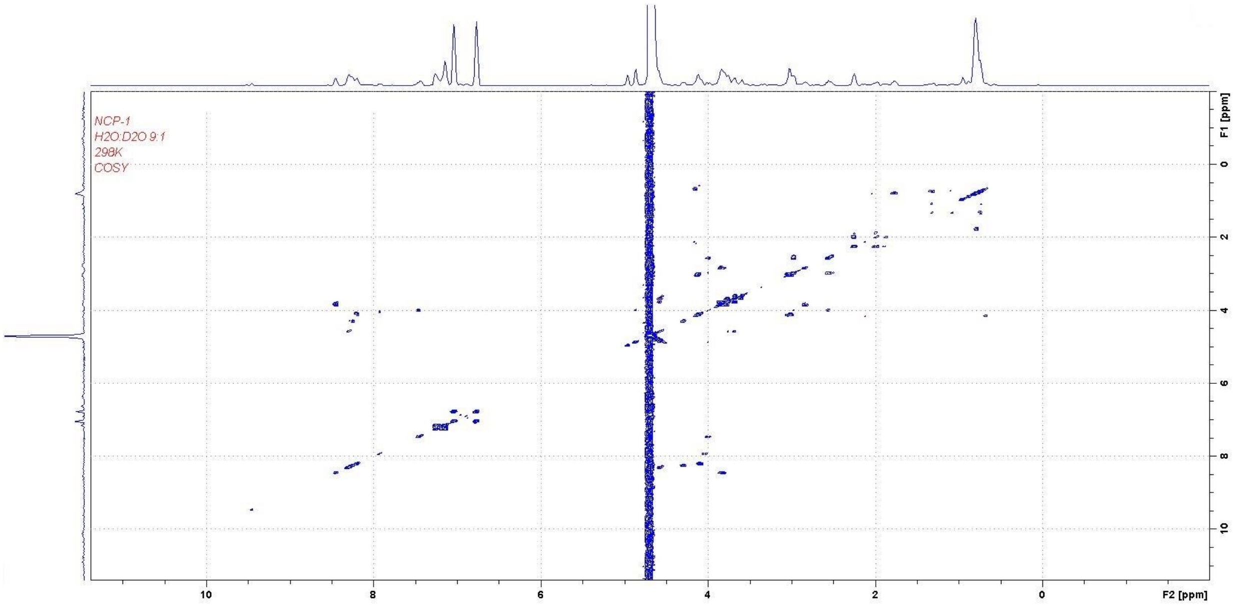

Figure S2: COSY spectrum of Ncp-A2-L in H_2O:D_2O (9:1)

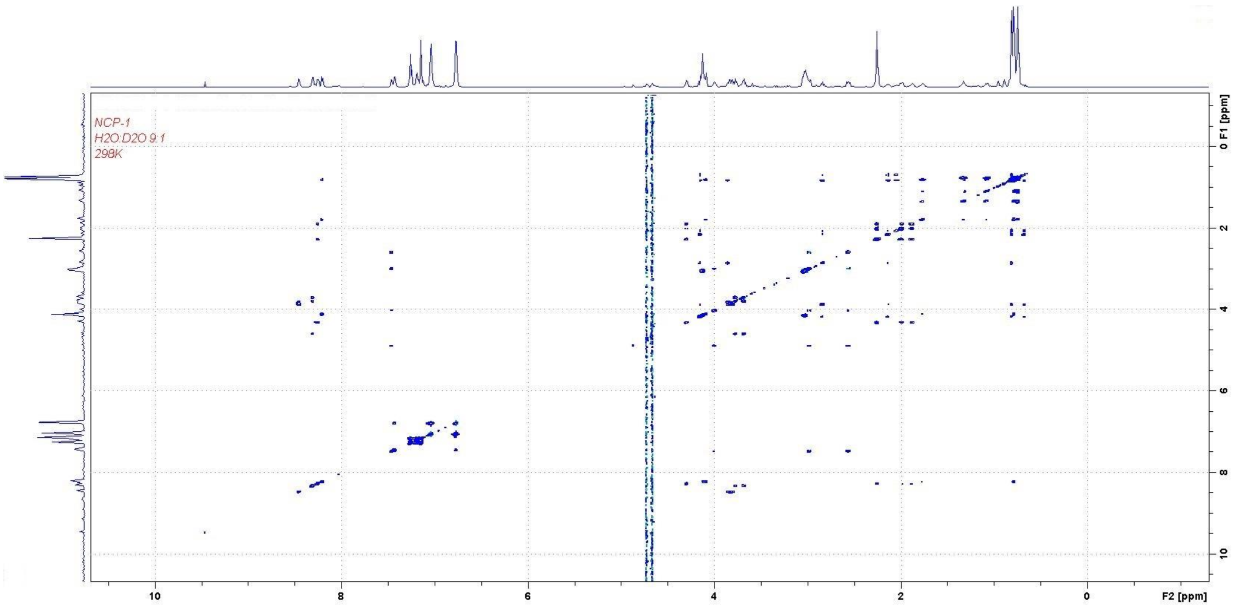

Figure S3: TOCSY spectrum of Ncp-A2-L in H_2O:D_2O (9:1)

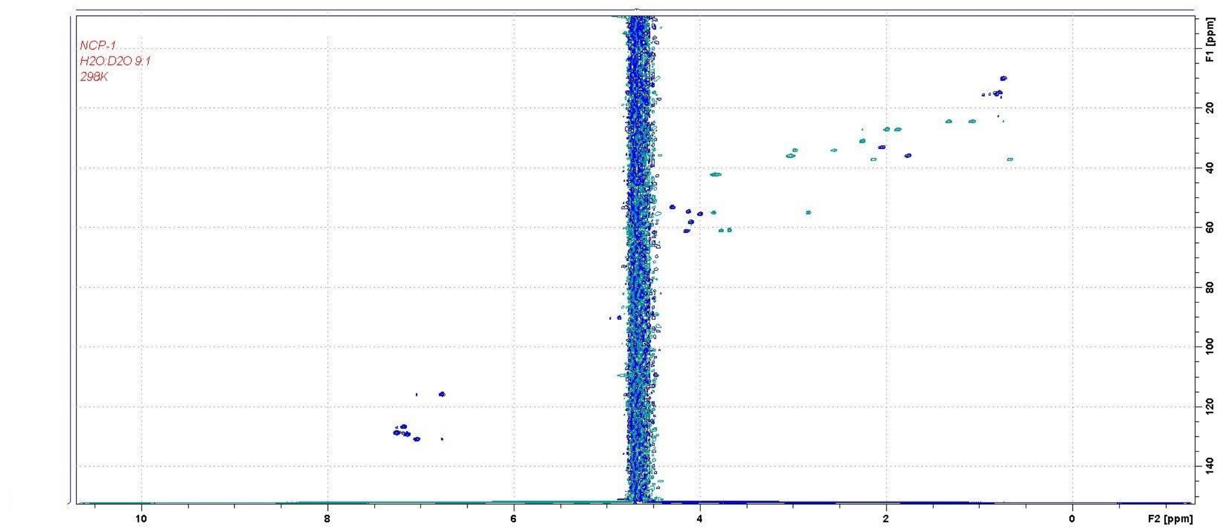

Figure S4: HSQC spectrum of Ncp-A2-L in H_2O:D_2O (9:1)

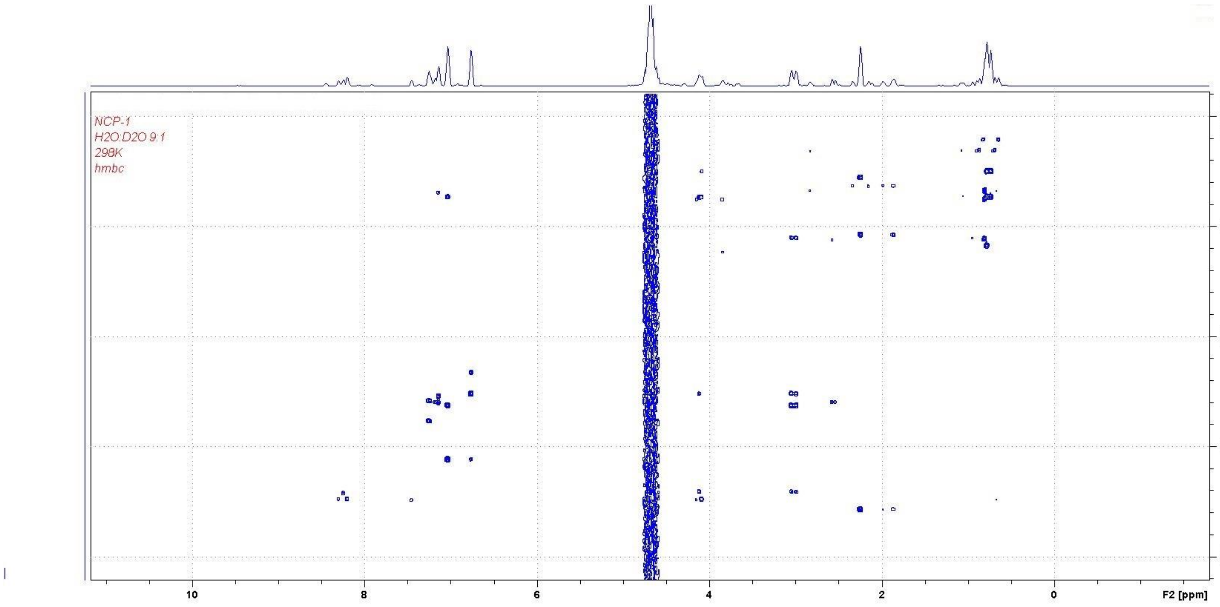

Figure S5: HMBC spectrum of Ncp-A2-L in H_2O:D_2O (9:1)
